# William Henry Mason

**Published:** 1918-10

**Authors:** 


**William Henry Mason,** Stockton, Calif.; Albany (N. Y.) Medical College, 1911; aged 33; radiologist at the Stockton State Hospital; died at his home in that institution, Aug. 20.

